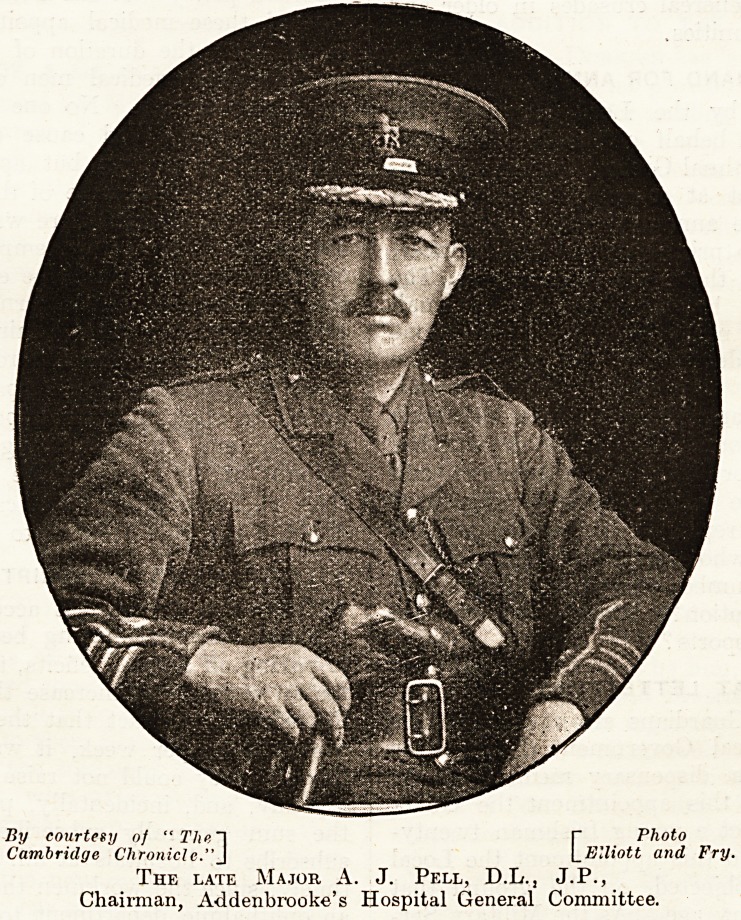# Hospital and Institutional News

**Published:** 1916-09-09

**Authors:** 


### HOSPITAL AND INSTITUTIONAL NEWS.

### THE LATE MAJOR PELL.

Last week we recorded the sudden death of Major A. J. Pell, chairman of the General Committee of Addenbrooke's Hospital. This office he had held for twelve years, and we briefly sketched the important movements and changes which had been effected under his chairmanship. To that brief notice we may add that Major Pell's election proved to be one of the most fortunate and important events in the history of Addenbrooke's. In several respects when he took the chair the hospital buildings were defective, and to-day, owing to the vigour introduced into the management, the untiring energy devoted to the raising of the necessary funds, and the business aptitude and skill which characterised the selection of the architect and the carrying out of the really excellent additions to and reconstruction of the buildings, there is ample evidence of the value to the sick poor of Cambridge, the county, and the Isle of Ely, of Major Pell's personlity and his association with this hospital. No one knows better the precise accuracy of this testimony than Mr. Richard J. Coles, the energetic secretary - superintendent of Addenbrooke's Hospital, who was appointed by Major Pell in 1904, and worked under him for twelve consecutive years. Major Pell proved to be one of the most capable and most businesslike chairmen in the hospital world of Great Britain, and his just and energetic government of his hospital proved a source of strength, the effects of which are likely to continue for many years forward. A memorial service was held in Ely Cathedral on Friday, September 1, in which the great body of the clergy took part. The large attendance was widely representative of the municipal and county governing authorities, of local society, and of all classes of the population. We have not space to give the names, but a study of them as they appear in the local paper demonstrates the deep affection and sympathy in which the late Major Pell was held, and the wide influence which his life-work and character exercised during his life on the whole community which he served so unselfishly, continuously, and well.

*By courtesy of "The Cambridge Chronicle."* *Photo Elliott and Fry.*

The late Major A. J. Pell, D.L., J.P., Chairman, Addenbrooke's Hospital General Committee.

### THE RESPONSIBILITY OF THE CRITIC.

Mr. Stephen Coleridge, even in war-time, wastes much energy in teaching his betters how to do it. His latest exhibition of the absence of a sense of responsibility adequate to the position he assumes as a critic is his reference to our obituary notice of Sir Victor Horsley (The Hospital, July 29, p. 409). He declares: "It would have been more to the purpose as regards the nurses, and a more manly part for The Hospital to play, if it had publicly rebuked Sir Victor Horsley for this alleged absolute brutality when he could have replied." On reference to The Hospital of August 1, 1914, page 492, he will find an article in which we reminded Sir Victor Horsley and others that their treatment of their nurses should be ended or mended. We cannot pursue the matter, and we credit Mr. Stephen Coleridge with every gentleman's view, *de mortuis nihil nisi bonum.*

### THE CONDITIONS OF SUPPLY OF SALVARSAN.

The Local Government Board have issued a circular indicating the conditions under which salvarsan or approved substitutes for it will be supplied free for intravenous injection in the treatment of syphilis. It is recommended that the medical officer of health should distribute the drugs, and that he should be required to satisfy himself, before issuing a supply, that the applicant is a registered practitioner with one or other of certain extra qualifications. These extra qualifications are left rather vaguely defined, and appear to present little guarantee that their possessor is really fit to be entrusted with the administration of these potent drugs; there are too many loopholes for "eyewash" and influence, as opposed to real knowledge and efficiency. The *Times* describes the scheme as a compromise, but thinks it better than might have been expected. Very possibly it may work all right, as so many other logically indefensible compromises do in this country. While on this topic, it is worthy of note that the Government of Victoria has introduced a Bill providing for the compulsory notification by doctors of all cases of venereal disease, and making it an offence for any person so suffering to infect another person under a penalty of £50 or twelve months' imprisonment. They are nothing if not thoroughgoing in these new countries, and if this Bill passes into law its effects will be of the greatest value as a guide to the framers of anti-venereal crusades in older and more cautious communities.

### THE EFFECTIVE DEMAND FOR ANNUAL REPORTS.

The inauguration by the Lord Mayor of the appeal for £5,000 on behalf of the Queen's Hospital for Children, Bethnal Green, has been accompanied by an attempt at economy in connection with the issue of the annual report. The committee has decided to print only a small number, and to dispense with the usual distribution. The economy represented by printing only a small number of copies is not great, for, as everyone knows, the real cost depends on the extent of the matter to be printed. The best method of curtailing the size of the annual report is a debatable one, and centres round the question whether scientific statistics or lists of subscribers and donors can the more conveniently be dispensed with. Copies of the report for inspection will be forwarded to those who apply for them. What proportion does the number of such applicants bear to the normal distribution? What is the effective demand for annual reports?

### LOYALTY AT LETTERKENNY.

The Letterkenny Guardians are at loggerheads with the (Irish) Local Government Board over the appointment of a dispensary medical officer. To fill a vacancy in this appointment the Guardians proposed to elect a young Irishman twenty-two years of age. To this appointment the Local Government Board objected, on the ground that the doctor is of military age. As the Military Service Acts do not apply to Ireland, this action of the Local Government Board shows a degree of backbone until lately lacking in Irish government. At the next meeting of the Letterkenny Guardians it was proposed that they should stick to their appointment, in spite of the objection from Dublin. This was carried, and it remains to be seen what the effect upon the Local Government Board will be. The simplest way out of the difficulty would be for the doctor in question to join the R.A.M.C.; failing such a step, it becomes a question whether the Central Medical War Committee or the British Medical Association should not take some notice of this and similar cases, if any there be. Arrangements whereby such medical officers should become permanently ineligible for all medical and institutional appointments in Great Britain and in the loyalist province of Ireland should not be hard to devise, and might do a lot of good.

### THE IRISH LOCAL GOVERNMENT BOARD'S FIRM POLICY.

Letterkenny is not the only place in Ireland where the Irish Local Government Board refuses to sanction a public appointment being given to a doctor of military age. A similar situation has arisen at Omagh; and in communicating the refusal to confirm the appointment as workhouse doctor of a man of military age the Local Government Board has defined its policy in clear and uncompromising terms. It is the "settled policy" of the Board not to countenance any of these medical appointments for a longer period than the duration of the war; and whenever possible medical men of over military age are to be chosen. No one who believes in the justice of the Allied cause can do anything but applaud this policy; but apparently it does not commend itself to some of the local authorities in Ireland. At Omagh there was already a doctor of over military age acting temporarily and willing to continue doing so until the end of the war. But the Guardians desired to turn him out and to elect as a permanency their Chairman's son. To this the Local Government Board objects because the latter is of military age. Apparently the Guardians intend to stick to their appointment, which may possibly end in their being surcharged for the amount of his salary. The issue is plainer even than at Letterkenny, and can hardly remain in doubt if the Board sticks to its principles.

### WORKMEN'S SUBSCRIPTIONS IN WALES.

The sums on deposit account at the Merthyr General Hospital having been steadily absorbed in paying off annual deficits, the time lately arrived for an attempt to increase the hospital's income. Considering the fact that the workmen contribute one farthing per week, it was determined to ask them if they could not raise their contribution to a penny, and, incidentally, place it on a par with the sum generally subscribed by workmen who subscribe to hospitals at all. The suggestion was timely, since the workmen themselves have desired an ophthalmic department to be established, have felt the hospital to be too small and the waiting-list often too long, and have also regretted that certain cases, after preliminary treatment, have to be sent on to Cardiff. The result is that some of the workmen have already adopted a penny-a-week scheme, but they have paid half of it to Cardiff. The case, therefore, for an increased subscription and the early extension of the hospital seems to be abundantly clear. The workmen's representatives have accordingly agreed to the appointment of a joint committee to consider ways and means. There is no reason why the Merthyr General

Hospital should not be as efficient and self-supporting as the workmen choose to make it. A "farthing contribution" and a "general hospital limited to giving preliminary treatment" are alike phrases which the men and their institution have outgrown.

### AN OBJECTION TO WOMEN CHAUFFEURS.

A doctor, applying for exemption for his chauffeur—aged thirty-nine and passed for general service—told the Birmingham Tribunal that women chauffeurs could drive all right, but the fatal objection to them was that they could not start the car! This particular man was physically sound, but constitutionally weak, which the Army would probably find out later. He had been in his employment eight years, and was at the present time doing the work of several other doctors as well. Agreeing that the chauffeur was doing useful work, the Tribunal granted three months' exemption.

### THE EXEMPTION OF A MEDICAL STUDENT.

At the Dudley Tribunal the Military Representative applied for the review of an exemption certificate granted to a young medical assistant at the Guest Hospital. It was stated by Mr. A. Bird, the secretary of the institution, that the appellant was the junior of two house surgeons, and had to sit for his final examination. Extraordinary difficulty was being experienced in securing house surgeons at present, and every day the position became more acute. Eventually the application of the military authorities was withdrawn upon certain conditions.

### A NEW MORTUARY CHAPEL.

A new mortuary chapel has been dedicated by the Bishop of Crediton at the South Devon and East Cornwall Hospital, Plymouth. In the past certain hospitals have failed to understand that the hospital standard must be maintained in the mortuary as highly as anywhere else, and that the hospital as a centre of education in the district which it serves is as much bound to inculcate respect for the dead, and consideration for bereaved relatives and friends, as it is to show sympathy for patients and kindness to their friends. It is therefore noteworthy that the initiative in the present case came from a lady, who, after visiting the hospital, offered a substantial sum on condition that the total required was subscribed in a given time. The result at last is the new mortuary chapel, in which Dr. Woollcombe has given the altar of carved oak. Oak has been freely used in the chancel—the panelled and crested reredos, the cross and candlesticks, and the bier are all of oak. The wall hangings and altar wings are of violet. These decorations and the furnishings generally have been given by friends, and the mortuary chapel is a worthy and most desirable addition to the institution.

### THE WAR AND POOR-LAW CENTRALISATION.

More than passing interest attaches to the movement now proceeding in North Essex for the closing of workhouses owing to the diminution in the number of inmates. At Dunmow there are only 102, though accommodation exists for 500; the same proportional fall exists, on a smaller scale, at Halstead, Braintree, and Lexden. The Dunmow Guardians therefore have decided to offer to receive all the Halstead inmates so as to allow the smaller institution to be closed; and a meeting of adjoining Poor-Law Unions is to be called to consider the possibility of further economy by combination. While not forgetting the interests of the staffs which may be affected in the case of the closed institutions, it is permissible to hope that the lessons in the value of combination now being learnt in time of war may not be forgotten when peace is declared; and a new step towards centralisation may now be beginning which should have a permanent and valuable effect on the future of Poor-Law administration.

### AN ADDITION TO THE WARD-LOCKER.

Lady Smith-Dorrien has issued a new appeal on behalf of her fund for providing soldiers in hospital with hospital bags for holding their personal things. Their usefulness, she adds, has been abundantly proved, and the bags must be classed as a substitute or addendum to the ward-locker which prevails at home. It is only war conditions which have suggested an alternative to the locker in the bag that can be dispatched after patients wherever they go.

### A PRACTISED DESIGNER OF HOSPITAL TABLEAUX.

The voluntary system produces so much hard individual effort, and gives opportunity to skill and talent in so many forms, that it is perhaps hardly surprising for the Royal West Sussex Hospital to have come to depend on one man for the invention and organisation of the tableaux which contribute to the annual demonstration. For eighteen years Mr. Oswald A. Bridges, the Bognor surveyor, has organised the hospital procession, and on the recent occasion almost every one of the tableaux was designed and invented by him. Since some of them had a topical air, and illustrated the war or military matters, both ingenuity and skill had plenty of scope. It is a remarkable instance of the way in which voluntary work persists, and both the hospital and Mr. Bridges may be proud of a record which does credit to them both.

### THE HOLDING-UP OF SANATORIA BUILDINGS.

One of the institutions which has experienced considerable difficulties owing to the war is the Liverpool Hospital for Consumption and Diseases of the Chest, with the associated sanatorium at Kingswood, Delamere Forest. It has been found impossible to complete the building of the new sanatorium at Fazakerley. The hospital itself has been full all the year, and the waiting-list does not readily diminish. The vast majority of patients come from Liverpool. The staff difficulty has been acute owing to the number of voluntary workers whom the demand for medical officers at the Front has taken away. Probably the one type of hospital construction which has suffered most from interruption through the war has been that of sanatorium buildings, and those who look ahead cannot be certain that the good work done before the war in educating public opinion to undertake preventive work against the disease will continue to find ready listeners when the host of problems created by a return to peace conditions presses upon the nation.

### HOSPITAL BENEFITS BY INQUEST FEES.

At a recent inquest at Bury St. Edmunds on a patient who died under anæsthesia at the West Suffolk Hospital, the jury devoted their fees to the funds of the hospital. This graceful act may be taken as an indication, which is fully borne out by the report of the evidence, that the jury were fully satisfied as to the correctness of the proceedings at the hospital and the blamelessness of the medical staff for the unfortunate fatality. As a matter of fact, the patient was suffering from some form of septic meningitis, originating in septic teeth: the operation was attempted as a last desperate chance, for the patient was already moribund. So it is not surprising that, with this serious intracranial disease, the anæsthetic should have proved too much for him. So long as surgeons are willing to take risks on the chance of saving life in an otherwise desperate case, so long will occasional misadventures of this sort take place. But it is not often that these incidents bring actual financial advantage to the institution concerned.

### THE 4TH SOUTHERN HOSPITAL'S "GAZETTE" AT PLYMOUTH.

There are no heavy articles in the August number of the 4th Southern General Hospital *Gazette*, but it is full of light reading, and contains a brief description of the country house on the moors to which fifteen nerve patients are sent for treatment. This is one of the younger *Gazettes*, being in its fifth issue, and consequently only now developing its character and style. Apparently illustrations are not very numerous, but experience shows that if they can be found, a series of thumbnail drawings inset in the text, though not necessarily illustrating it, give a varied air to the pages which a smaller number of larger drawings often fails to do. The advertisements, by the way, include one of the Royal National Pension Fund for Nurses, which, if we remember, has not taken advantage of the *Gazettes* in a similar manner before.

### A "GAZETTE'S" FIRST BIRTHDAY.

The first birthday of the *Gazette* of the 3rd London General Hospital, Wandsworth, has now taken place, and a new volume will begin next month. The paper may congratulate itself upon the fact that during the past year only three articles, three poems, and two sketches from outsiders have been printed. The *Gazette* is fortunate in retaining contributors who have left the hospital. Thus Mr. C. R. W. Nevinson has sent a futurist drawing entitled "The Sprucers," which is reminiscent of the early days when the blocks were still being equipped, and backs "intended for light articles only" were weary and asleep on heavy bales marked "Urgent" as in a nightmare. There are numerous articles, photographs, and drawings in the September number, which, as usual, distils the humour to be found in the daily life of staff and patients.

### NURSES' RECREATION AT HULL.

One of the Hull Guardians, a Mr. Councillor Fussey, is not going the right way to achieve popularity in nursing circles. It is little that can be done to provide means for the recreation of nurses in an infirmary, but when that little can be brought about without undue expenditure of public money there are few that would join with Mr. Fussey in making a fuss. It seems that the House Committee of the infirmary recommended the modest outlay of £6 on the laying out of a tennis-court for the use of the officers and nurses. Mr. Councillor Fussey made a strong protest, and, developing his objection, drew a would-be harrowing picture of patients dying while the nurses and staff were absorbed in their game. Another Guardian seconded rejection on the quite legitimate grounds that an open space provided for the use of the inmates generally would be curtailed. Other Guardians pointed out that nurses are very difficult to get and need all the encouragement that can be given them. Mr. Fussey, with an inconsistency which may not be unexpected, said that the nurses could play tennis at some neighbouring courts, quite forgetting his previously expressed desire that they should always be within call. Fortunately, the recommendation of the House Committee was carried, with only two dissentients.

### THIS WEEK'S DRUG MARKET.

The downward tendency of prices continues to make buyers very cautious, and, apart from the steady business that is being done to meet the needs of the Government, transactions are confined to very small quantities. The lower price of aniline oil has led to a further reduction in quotations for acetanilide, which drug is now more freely available. Aspirin is again lower, and salicylic acid and salicylate of soda are continuing to tend downwards in value; the salicylates, however, are still something like nine times the pre-war value, although they are only half the price quoted at the beginning of this year. Phenacetin is scarcer than ever, and there are no signs that the supply will be more plentiful in the immediate future. Bromides are unchanged. Cocaine is slightly lower, but the decline in value has not so far been very pronounced. Potassium permanganate seems to be in rather better supply, and the price is slightly lower. Following on the reduction in price of citric acid, makers of potassium citrate, sodium citrate, and iron and ammonium citrate have lowered their quotations. English growers of lavender are disappointed with the yield of oil, which has not come up to expectations.

## Figures and Tables

**Figure f1:**